# Intra-Ocular Circulation; Rhythmical Changes in the Venous Pulse of the Optic Disk

**Published:** 1878-11

**Authors:** O. F. Wadsworth, James J. Putnam

**Affiliations:** Boston, Mass.; Boston, Mass.


### INTRA-OCULAR CIRCULATION: RHYTHMICAL CHANGES IN THE VENOUS PULSE OF THE OPTIC DISK.

By DR. O. F. WADSWORTH and DR. JAMES J. PUTNAM, of Boston, Mass.

The investigation to be described in this paper was undertaken by us originally for the purpose of studying for ourselves the effects of compression of the veins and arteries of the neck, and of inhalation of amyl nitrite on the retinal circulation. It is published, however, mainly for the sake of the observations whose nature is indicated in the title of this paper; and our results as regards the other points, which have so often been the subject of controversy, will be only briefly noted.

The method of experimentation was always the same, one of us always examining by the upright method the same eye of the other, the pupil fully dilated with atropine. To further fix the appearances a careful sketch of the disk and vessels was made.

It was soon found that the best point on which to fix attention was a main branch of the vena centralis, which pulsated distinctly as it turned to plunge into the physiological depression near the centre of the disk, receiving at its bend a smaller branch which also pulsated. Changes in the size and shape of the pulsating portion of these vessels formed the most obvious, indeed the only reliable signs of variation in the vascular supply to the fundus.

1. *Compression of the Jugular Veins.*—This was done by twisting a handkerchief about the neck. The compression could easily be carried so far as to cause the veins to stand out in relief on the forehead, and the whole face to assume a dusky hue, not to speak of causing blurred vision and an uncomfortable sense of fullness in the head.

The effect of this procedure on the intra-ocular circulation was, however, so slight, if there was indeed any, as not to be made out with certainty. Sometimes an appearance of greater fullness of the veins at the pulsating point seemed to be present, but the pulsations went on as before.

2. The effect of the *pressure on the carotid* of the same side with the eye examined, were studied in the following manner: The pressure was made at about one-and-a-half inches above the clavicle by one finger of the examiner, and was increased to a degree sufficient both to stop the beating of the temporal artery, and to cause a distinct sensation in the head, quite different, it may be said, from that due to compression of the jugular veins. So soon as this result was produced all pulsation in the vein ceased abruptly, and the vein remained narrow, as in the condition of so-called venous systole, so long as the pressure was kept up. Immediately on relaxation of the pressure on the carotid the vein refilled completely, and the normal pulsation was re-established.

This observation seemed to us to have an important bearing on the theory of the venous pulse. It is well known that two prominent theories with regard to this phenomenon are held; one, that of Coccius, who maintained that at the time of arterial diastole the blood flowed out of the eye, through the veins, more rapidly than before, the veins at the same time partially collapsing near their point of exit; the other, that of Donders, according to which, at the time of arterial diastole, the vein at its point of exit is compressed, which causes a backing-up of the blood just behind this point. The latter view is the one which appears supported by the results of the experiments just described. Here the pressure on the carotid prevented the arterial diastole, and so long as the pressure was kept up that part of the vein which normally pulsated remained in a semi-collapsed condition, *i. e.*, in the condition of so-called venous systole.

So soon, however, as the pressure was removed, and the heart's impulse again allowed to distend the artery, this portion of the vein refilled, entering into the condition of venous diastole, and then the pulsation went on as before.

3. *Inhalation of Amyl Nitrite.* The *first* result of two or three full inhalations of this drug, poured upon a handkerchief, was that at once, even before the full sensorial effect had developed itself, the vein at the pulsating point became reduced in size, as it had done when pressure was made on the carotid; the pulsation did not, however, as in that case, cease altogether, but became rapid, and sometimes imperceptible, always slight. No change could be detected in the appearance of the arteries at this stage, nor of the veins, except at the point referred to.

As a *secondary* result of the inhalations, which made its appearance just about the time that the sensorial symptoms began to abate, the veins refilled at the pulsating point, the pulsations themselves becoming again more manifest. A more exact account of the condition of the vein at this period will be given after the description of the rhythmical changes alluded to at the outset of the paper.

4. *Rythmical Changes in the Venous Pulse.* When the portion of the veins above referred to was attentively watched, it was seen that besides the changes which have been recognized as constituting the venous pulse, there were other periodical variations in the size of the pulsating portion of the vessel. These latter occurred at intervals which, by their length, recalled the rhythmical changes in arterial tension, described by Traube, Hering,

Cyon, and Sigmund Mayer, and which perhaps are the cause of the long waves of movement of the brain, noticed by Mosso and others, including one of ourselves. In other words, besides pulsating in the usual manner, the vein, at the point alluded to, was seen to dilate and contract gradually, in periods corresponding to about five respirations. The vein thus seemed to pulsate under the influence of two distinct systems of waves, one synchronous with the cardiac impulses, the other, the long waves, due perhaps to changes in arterial tension. Under the influence of these long waves the diameter of the vein varied, independently of the ordinary pulsations, often as much as in the proportion of two to one.

While such a wave was at its height, each ordinary pulsation seemed to diminish the diameter of the vein by about one-half, whereas as the lowest point of the wave, ordinary pulsations almost obliterated the calibre of the vein.

The passage from the highest to the lowest point of these long waves was gradual and pretty regular, though not absolutely so, the period of each wave occupying, as has been said, the time of about five respirations.

These waves were to be made out at every examination, when carefully sought for, but were not always equally marked.

To recur now to the condition of the circulation during the second stage of the amyl experiments, when, as was said, the vein refilled, as the subjective symptoms began to diminish in intensity. It was noted that after refilling, the vein remained for a few seconds persistently distended to the size which it usually had at the height of the long waves, the cardiac pulsation continuing however, and then the long waves again began to show themselves.

In the first two experiments with amyl nitrite the period of inhalation was short, and it occurred to us as a possibility that the coincidence of the secondary filling of the vein with the passing off of the subjective symptoms might be only accidental. To determine this point the inhalation was repeated, and continued a much longer time than before.

In this instance, also, the vein remained narrowed as long as the effect of the drug was kept up, and again refilled as the subjective symptoms diminished. During this last experiment it did appear as if there were some slight increased fullness of all the veins, but it was, at the most, of trifling amount. No change in the general color of the disk was observed in this or any other experiment. *Journal of Nervous and Mental Diseases.*

